# African Histoplasmosis in HIV-Negative Patients, Kimpese, Democratic Republic of the Congo

**DOI:** 10.3201/eid2411.180236

**Published:** 2018-11

**Authors:** Nestor Pakasa, Asaf Biber, Samuel Nsiangana, Désiré Imposo, Ernest Sumaili, Hypolite Muhindo, Maria J. Buitrago, Iris Barshack, Eli Schwartz

**Affiliations:** University of Kinshasa Hospital, Kinshasa, Democratic Republic of the Congo (N. Pakasa, E. Sumaili, H. Muhindo);; Chaim Sheba Medical Center, Ramat Gan, Israel (A. Biber, I. Barshack, E. Schwartz);; Institut Médical Evangélique, Kimpese, Democratic Republic of the Congo (S. Nsiangana, D. Imposo);; University of Antwerp, Antwerp, Belgium (H. Muhindo);; Instituto de Salud Carlos III, Madrid, Spain (M.J. Buitrago)

**Keywords:** African histoplasmosis, Histoplasma capsulatum var. duboisii, Democratic Republic of the Congo, Kimpese, fungi, histoplasmosis

## Abstract

We describe a case series of histoplasmosis caused by *Histoplasma capsulatum* var. *duboisii* during July 2011–January 2014 in Kimpese, Democratic Republic of the Congo. Cases were confirmed by histopathology, immunohistochemistry, and reverse transcription PCR. All patients were HIV negative. Putative sources for the pathogen were cellar bats and guano fertilizer exploitation.

Infection with the fungus *Histoplasma capsulatum* var. *duboisii*, also known as African histoplasmosis, was described by Dubois et al. in 1952 (*1*). In total, <300 cases have been reported in the literature in Africa, mostly in sporadic forms (*2**,**3*).

Although the classical histoplasmosis, caused by *H. capsulatum* var. *capsulatum*, involves mainly the lungs, African histoplasmosis commonly involves the skin, followed by the bones. It tends to occur more frequently in patients infected with HIV. The pathogenesis of classical histoplasmosis, inhaling spores from bats’ and birds’ soil or guano, is well established, but the pathogenesis of African histoplasmosis remains unclear (*2*).

In the past, few reported cases of African histoplasmosis have been described from the Democratic Republic of the Congo; all were sporadic (*3**–**6*). We describe an unusual case series of African histoplasmosis in HIV-negative patients in Kimpese, Democratic Republic of the Congo.

## The Study

All tissue samples diagnosed from histopathology as African histoplasmosis in routine biopsies at Institut Médical Evangélique Kimpese (Kimpese) during 2011–2016 were included in the study. Most patients were female; median age was 20.5 years, and 42% percent were school-age children (Table). All but 3 were residents of Kimpese. Most of the infections occurred during July 2011–October 2012 (n = 32; 88.9%); case rates then sharply declined in 2013 (n = 3; 8.3%), subsiding to zero after January 2014 (Table). Four patients who were available for interviews reported living in houses that were heavily infested with cellar bats; these persons frequently collected guano from cellars to fertilize gardens, although their official occupation was not agricultural.

**Table:** Characteristics of patients with African histoplasmosis, Democratic Republic of the Congo, July 2011–January 2014

Characteristics	Value*
Sex	
M	13 (36.1)
F	23 (63.9)
Year of diagnosis	
2011	13 (36.1)
2012	19 (52.8)
2013	3 (8.3)
2014	1 (2.8)
Age, y, median (interquartile range)	20.5 (10.5−39.0)
3–16	15 (41.7)
17–49	15 (41.7)
>50	6 (16.6)

*Values are no. (%) except as indicated.

Laboratory findings, mainly from blood and feces, were nonspecific, apart from an increased erythrocyte sedimentation rate in most patients, a feature not diagnostic per se in the tropics. HIV test results were negative for all patients.

A total of 36 consecutive routine biopsies yielded diagnoses of *H. duboisii*. Seven specimens were from skin, 7 from bones, 5 from lymph nodes, and 8 of crumbly necrotic material. The rest of the specimens were labeled tumor or tumefaction related to clinical preoperative diagnosis. 

Biopsy samples were fixed in 10% formalin and processed in the local laboratory using standard techniques of hematoxylin and eosin (HE) staining for light microscopy. Because special staining for fungi is unavailable in Kimpese, paraffin blocks were sent to Sheba Medical Center (Ramat Gan, Israel), where control HE, periodic acid Schiff (PAS), and Grocott methenamine-silver (GMS) staining were performed. Paraffin blocks were also brought to the Institut Pasteur Paris (Paris, France) for immunohistochemistry (IHC) to confirm the identity of the fungus using a noncommercial monoclonal antibody that detects both *H. capsulatum* and *H. duboisii*, distinguishable by their respective sizes (1–5 μm vs. 7–15 μm in diameter). The fungus phenotype was finally validated as *H. capsulatum* var. *duboisii* by the referral center le Centre National de Référence des Mycoses Invasives et Antifongiques in Paris. In addition, molecular analyses were performed at the Mycology Reference Laboratory, Centro Nacional de Microbiología, Instituto de Salud Carlos III (Majadahonda–Madrid, Spain), using a multiplex in-house specific real-time reverse transcription PCR (RT-PCR), as described previously (*7*).

In all tested samples, many intracellular or extracellular microorganisms were conspicuous on HE sections (Figure, panel A). In most cases, organisms were seen in the cytoplasm of multiple multinucleated Langhans-type giant cells, often dividing by explosive budding (Figure, panel A) and frequently demonstrating explosive giant asteroid bodies (Figure, panel B), at times undergoing degeneration. The fungus was easily identified on PAS (Figure, panel C) and GMS (Figure, panel D).

**Figure:**
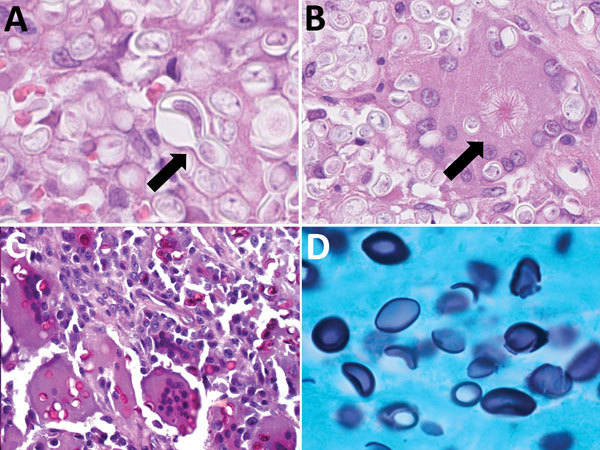
Pathologic findings from patients infected with African histoplasmosis, Democratic Republic of the Congo, July 2011–January 2014. A) Yeast explosive budding (arrow) (hematoxylin and eosin [HE] staining; original magnification ×160); B) asteroid bodies (arrow) (HE staining; original magnification ×160); C) yeasts in Langhans cells (periodic acid Schiff staining; original magnification ×160); D) lemon-shaped appearance (Grocott methenamine-silver staining; original magnification ×80).

Twelve samples from different patients underwent further IHC staining, which revealed a membranous staining of large 7–15-μm yeasts. RT-PCR assays performed in paraffin-embedded tissue samples from 3 patients were all positive for *H. capsulatum*. The technique was unable to differentiate between *H. capsulatum* var. *capsulatum* and var. *duboisii*, because the specific probe was designed to detect both. The average fungal burden detected was 7.6 fg DNA/μL.

## Conclusions

This histology-based study identified a novel focus of *H. duboisii* in the Democratic Republic of the Congo in the city of Kimpese and its vicinity. Over a period of ≈2 years, 36 cases were routinely detected in a single pathology laboratory in this area; all patients were HIV negative.

The putative source of infection appears to be cellar bats, *Chaerephon pumilus*, and guano fertilizer overexploitation. African histoplasmosis has previously been reported to be isolated from the intestinal contents of bats belonging to the species *Nycteris hispida* and *Tadarida pumila* from a cave in rural Nigeria and from soil mixed with bat guano (*8*).

The subsiding of new cases could be related to the incidental cessation of the use of bat guano along with the reintroduction of chemical fertilizers. This change was unrelated to any knowledge about the infection risk of guano and may be reversed when the guano accumulates again to sizable amounts.

This case series featured a high incidence of infection in women and girls and in young children, including toddlers and school-age children. Six patients were 3–7 years of age, an age when children are more tied to their mothers, sit on the ground, and may be in contact with contaminated soil. Although HIV tests were negative, other innate or acquired immunosuppression was not ruled out.

During the first year of our case series, more cases were initially labeled as lymphoproliferative disorders or extrapulmonary tuberculosis, whereas in subsequent years, cases were initially suspected to be histoplasmosis infections. Therefore, it is reasonable to assume that, in endemic areas, where histopathological diagnosis or advanced microbiology labs are not widely accessible, the disease may be underdiagnosed; therefore, the prevalence of African histoplasmosis may be underestimated.

The diagnosis of African histoplasmosis is made mainly by histopathology, which shows granulomatous inflammation and giant cells containing numerous large yeast cells 10–15 μm in diameter, which are thick-walled and divided by narrow budding. The fungus is easily visible by GMS, Gridley, or PAS staining (*4*). IHC staining, which was performed on 12 specimens, is not specific for African histoplasmosis but to *H. capsulatum*; therefore, it may play a role in more accurate and faster diagnosis and enable exclusion of other fungal infections.

In our series, 3 samples were tested by RT-PCR and results were positive in all 3 for *H. capsulatum* diagnosis. Newer PCR protocols have been developed and may provide rapid and species-specific diagnosis (*9*).

Our histopathologic observation has some limitations. IHC and the RT-PCR primers that were used are unable to differentiate between *H. capsulatum* var. *capsulatum* and var. *duboisii*, yet they confirmed the histoplasmosis diagnosis, and IHC together with the large size of the yeast make it specific for *H.* *duboisii*. Soil and the bat guano from the infected areas have not yet been tested for *H. capsulatum* var. *duboisii*. Among the strengths of this study is that this unusual case series is unique in its extent and clustering of cases. In addition, the available data add novel epidemiologic and diagnostic information to the current knowledge about this neglected disease.

Further investigation should be conducted to understand the reservoir of the pathogen, the types of daily activities that might pose risk factors for acquiring the disease, and the mode of transmission and progression of the disease. Because diagnosis, especially in these rural areas, is challenging, seroepidemiologic surveys are needed to establish the extent of this infection.
